# Turning stories into learning journeys: the principles and methods of Immersive Education

**DOI:** 10.3389/fpsyg.2024.1471459

**Published:** 2024-12-06

**Authors:** Riccardo Brunetti, Silvia Ferrante, Anna Maria Avella, Allegra Indraccolo, Claudia Del Gatto

**Affiliations:** ^1^Experimental and Applied Psychology Laboratory, Department of Human Sciences, Università Europea di Roma, Rome, Italy; ^2^Project xx1, Rome, Italy; ^3^Department of Developmental Psychology and Educational Research, ‘Sapienza’ University of Rome, Rome, Italy

**Keywords:** narrative-based education, experiential learning, immersive theatre, psychological foundations, pedagogical theory, simulation

## Abstract

This paper describes the theoretical and practical aspects of Immersive Education, an educational methodology based on interactive narratives, articulated as emotional journeys, to develop competencies. It has been developed throughout three school years (2021–2024) with more than 400 students (8–12 years old) in Public Schools in Italy and Spain. Immersive Education can be integrated with curricular school activities and can be used to target both curricular and transversal learning objectives, specifically the ones connected with the Personal, Social and Learning to learn Key Competence (LifeComp European framework). The paper describes the inspirations that led to the creation of the methodology, including similar experiential learning approaches. It then analyses the theoretical principles of the methodology, dividing them in four key-concepts, along with psychological evidence supporting them. The four key-concepts describe how immersive education aims at being a motivation trigger, featuring a dramatic structure, how it is based on the involvement of the self, and how it focuses on fostering a continuous engagement. It continues with a detailed analysis of implementation strategies, specifically about the management of emotional triggers and reactions, enriched by numerous examples taken from the projects implemented with the students. The conclusions open the way to future research directions to measure the impact of this approach on the development of transversal and specific competences.

## 1 Introduction

Stories are ubiquitous in our daily life: most of the time we speak, perceive, read, and even think, we organise information in an ordered sequence. Such relevance has been the basis to consider narratives as one of the primary mechanisms to build and communicate meaning (Bruner, 1991, 2004). Some authors (Schank, 1982; Schank and Abelson, 1977) have proposed that a significant part of our knowledge about life situations and events is represented mentally in a story-like framework (e.g., a *script*). This knowledge is used to orient in known situations and to understand novel ones.

A ‘story’, intended as an ordered sequence of events, can be the basis for different ‘narratives’, how that specific sequence of events is presented (Abbott, 2020): different narratives are able to generate different meanings, even if based on the same story. The potential of narratives as teaching tools has been leveraged since the dawn of mankind. There are countless examples of the use of narratives in education, recent and old, for different kinds of learners: in science for youngsters (Prins et al., 2017), in foreign language learning for kids (Fojkar et al., 2013), across different subjects for convicts (Butcher, 2006), in morals for the general population (King, 2008; Potter, 2023), just to name a few examples of the pervasiveness of the phenomenon. Narratives provide an effective means to organise information, while stimulating the imagination of the listener. This increased engagement could generate the feeling of an ‘immersion’, not only in environments, conditions and situations, but in semantics, emotions, and activities. The connection between *experience* and *learning* is a central theme of what has been called the ‘Copernican revolution’ of education (Rosenthal, 2009), between the XIX and the XX centuries: the shift of the educational perspective from the focus on the teacher and the subject, to the focus on the learner. It is Dewey (1938/1963) pedagogical activism that will set the basis for such a revolution, setting its foundations in the simple but powerful idea that ‘all genuine education comes about through experience’ (p. 25).

More recently, the pedagogical reflection embraced the idea that teaching, instead of simply offering knowledge to learners or aiming at changing their behaviour, should facilitate the learning process by shaping the learners’ experience itself (Morris, 2020). This way of approaching the learning process appeared in synergy with other recent changes. The focus on competences (Le Boterf, 2000; McClelland, 1973), defined as an underlying set of personal characteristics that facilitate superior performance (e.g., Boyatzis, 1982, 2008; Wong, 2020) in specific domains, gradually gained momentum in the education system internationally, calling for a change in educational strategies to make them more experience-based. Another aspect of this approach was a change of focus, from teacher-or content-centred approaches to theories centred on the learner, pointing out how knowledge of the learning process itself may guide us towards better teaching strategies. Among all the theories linking experience to learning, one of the most influential is the *Experiential Learning Theory* (ELT) (Kolb, 1984, 2015). For the ELT, life experience is a central and necessary part of the learning process, where ‘*knowledge is created through the transformation of experience*’ (Kolb, 2015, p. 49; original emphasis).

The methodology presented here as *Immersive Education* is a direct expression of the synergy between many of the approaches briefly outlined above and is connected to what has been defined as *Immersive Learning*: ‘Immersive Learning is not simply ‘learning with immersive media’ but Immersive Learning means learning with artificial experiences’ (Dengel, 2022, p. 2). In the case of the Immersive Education methodology, these artificial experiences are not set up in virtual learning environments (e.g., VR, AR, XR, etc.), but in different natural spaces in the school (classrooms, halls, storage rooms, offices, etc.) turned, often thanks to a set-up, into settings for the development of the narrative. Technology can be certainly integrated in this process, but it is not a central feature of the methodology. Immersive Education is a term describing strategies to *facilitate learning through the interaction with a narrative where the students play a central active role*. More specifically, the term indicates several strategies used to give to the narrative a transmedial,^1^ lifelike, deeply interactive character, triggering agency in the students. In other words, Immersive Education is a rich and believable journey based on a narrative, calling for a direct and active participation from the pupils as if it was actually true. The account presented in this contribution is based on the application of Immersive Education in several projects, spanning three school years (2021–2022, 2022–2023, and 2023–2024) in Italy and Spain, for more than 400 hundred pupils, ranging from 8 to 12 years old.

The use of interactive narratives as a stimulus for experiential education aims at producing deeper, long-lasting learning outcomes, compared to more passive, traditional teaching methods. Both direct experience and narratives are able to create more connections to long-term meanings, also thanks to the structured involvement of emotions in the learning process. By using the joint potential of experiential learning and narratives, Immersive Education’s general goal is to reach more learners and generate better quality learning.

The rest of this paper will firstly examine the theatrical origin of the term ‘Immersive’; then it will delve in describing the four key-concepts guiding the methodology of Immersive Education; and finally will analyse several of the strategies used to design and implement an *Immersive Education Experience*, along with numerous examples.

## 2 Experiential theatres

The expression ‘Immersive Theatre’ has been used as an umbrella-term to describe several different forms of interactive theatre focused on audience agency and multisensory storytelling (Alston, 2013; White, 2012). While this term has been undergoing a commercial and artistic explosion in its use during the last 20 years, its exact definition has always been debated; Lewis and Bartley (2023) recently proposed to include Immersive theatre inside the broader category of *Experiential Theatre*.^2^ This latter term describes theatrical practices that focus on audience involvement, defined as interacting with the event through a fully embodied act of perception (Nelson, 2010) and where specific forms of agency are allowed (White, 2013). Experiential Theatres implement different strategies including, among others: multisensory engagement, participation, game-play, and role-play. This kind of theatre alters the traditional spectatorship conventions (e.g., sit quietly in a dim lit room, to listen and watch what is put in front of you, etc.), to stimulate different kinds of interaction with a performance, through specific operations of experiential design.

This approach, based on the idea of art as experience, developed throughout the XX century, stemming again from Dewey’s ideas (Dewey, 1934/1980). During the last 20 years there were numerous examples of these kinds of Experiential Theatres, ranging from Bond and Lloyd’s *You Me Bum Bum Train* (2004); through Punchdrunk’s *Sleep No More*, NYC (2011), *The Drowned Man* (2013) and *The Burnt City* (2022); to Enrique Vargas’ *El hilo de Ariadna* (many versions, from 1993 to 2018). All these productions feature multisensory storytelling and the possibility for the participant to interact with the performance itself, through different kinds of engagement. Audience participation has been described as a choice with aesthetic effects (White, 2013, p.40–44) and has been categorised into four main kinds: *overt* (e.g., addressing spectators directly, making it clear they are being asked to respond in some way), *implicit* (e.g., an open possibility for interaction without an explicit invitation, as in leaving a phone ringing without anyone answering), *covert* (e.g., when it is not clear for the participants that they are involved in a theatre play, as in Boal’s ‘Invisible Theatre’; Boal and Epstein, 1990), and *accidental* (e.g., when no invitation is present, but the spectators misread what is happening as one, or interject deliberately with a performance).

One of the common features of experiential theatres is the ‘immersion’ of the participants in the experience of participation itself, a concept that has been carefully shaped and touches different layers of meaning (see Punpeng and Yodnane, 2023). The kind of strategic planning and writing in these types of theatres is different from more traditional, non-interactive performances, and the role and training of the actors/performers should make them responsive to any interaction that may arise with the audience (Brunetti, 2017). The author of interactive narratives has been defined as a *procedural author* (Murray, 2017; White, 2013), namely an author who is able to create the conditions to welcome the potentially unknown contribution of the audience as a part of the art piece itself. The performers of experiential theatre must therefore be trained in a different way than traditional actors, as they must be able to embrace, stimulate, develop, and strategically hold in check audience participation (see Lewis and Bartley, 2023).

Experiential theatres expand the notion of spectatorship, along the lines suggested by Rancière (2009, p.3) (‘active participants as opposed to passive voyeurs’, *The emancipated spectator*) and Bourriaud (2002, p.99) (‘the beholder is the joint creator of the work’ *Relational aesthetics*; see also Machon, 2013), by focusing on audience *agency* (the ability to influence, change or impact the situation, Breel, 2023; see also Nelson, 2010). This focus allowed performances to venture into the territories of intimacy, immediacy, direct contact, multisensoriality (Machon, 2013), and caught the interest of large portions of spectators, all around the globe. The success of this kind of theatre has been previously linked to the necessity of contemporary audiences for ‘an antidote to the alienating experiences of globalisation and virtual socialising and networking’ (Machon, 2013, p. 121), to specific contemporary business practises or political values (Alston, 2013), or to the fact that participation is a crucial part of theatre practises since ancient times (Lewis and Bartley, 2023), just to name a few. What is certain is that audiences are now more used to browse the internet and actively look for information and entertainment, rather than passively receiving what is provided by a few outlets (e.g., tv channels, newspapers, etc.); they are used to play video games—an art form based on agency; they are constantly managing multiple communication streams through digital channels.

Any kind of artistic expression must take into account their target, and the contemporary target are the people living through our times (Brunetti and Ferrante, in press). Similarly, the target of any contemporary educational activity is a sample of a population with the same features (e.g., living through our current times), susceptible to the same experiential dynamics as the audiences that populate Immersive Theatre performances. The application of the Experiential Theatre *dispositive* (Agamben, 2006; Foucault, 2004) can be thus useful also in educational endeavours, with the objective of captivating attention and triggering active participation.

## 3 Immersive learning projects

The term *Immersive Learning* refers to an educational experience based on eliciting immersion by inviting participants in virtual or physical spaces (Dengel and Mägdefrau, 2018). When these spaces are based on narratives, the ‘immersion’ fosters diegetic concern within the contextual meanings and narrative and, when narratives are made interactive, facilitating the psychological absorption with the challenges and tasks (Nilsson et al., 2016). The idea of teaching through collective imaginary situations can be traced back to ‘playworld’, an aesthetic play pedagogy firstly proposed by Lindqvist (1995), and further developed, among others, by Fleer (2018) into ‘Conceptual Playworlds’.^3^

The use of interactive narratives for educational purposes has been experimented several times in schools, in the last years, in different countries. While a complete survey of such experiences falls outside the purposes of this paper, we may annoverate among these the educational projects proposed by the organisation known as Punchdrunk Enrichment (Higgin, 2018a; Osborne, 2023) in the United Kingdom; by the collective Coney, with their ‘Adventures in Learning’ (Bowtell, 2015) in the United Kingdom; and by the organisation Project xx1, in Italy and Spain. This paper is based on Project xx1’s projects and experiences.

**Figure 1:**
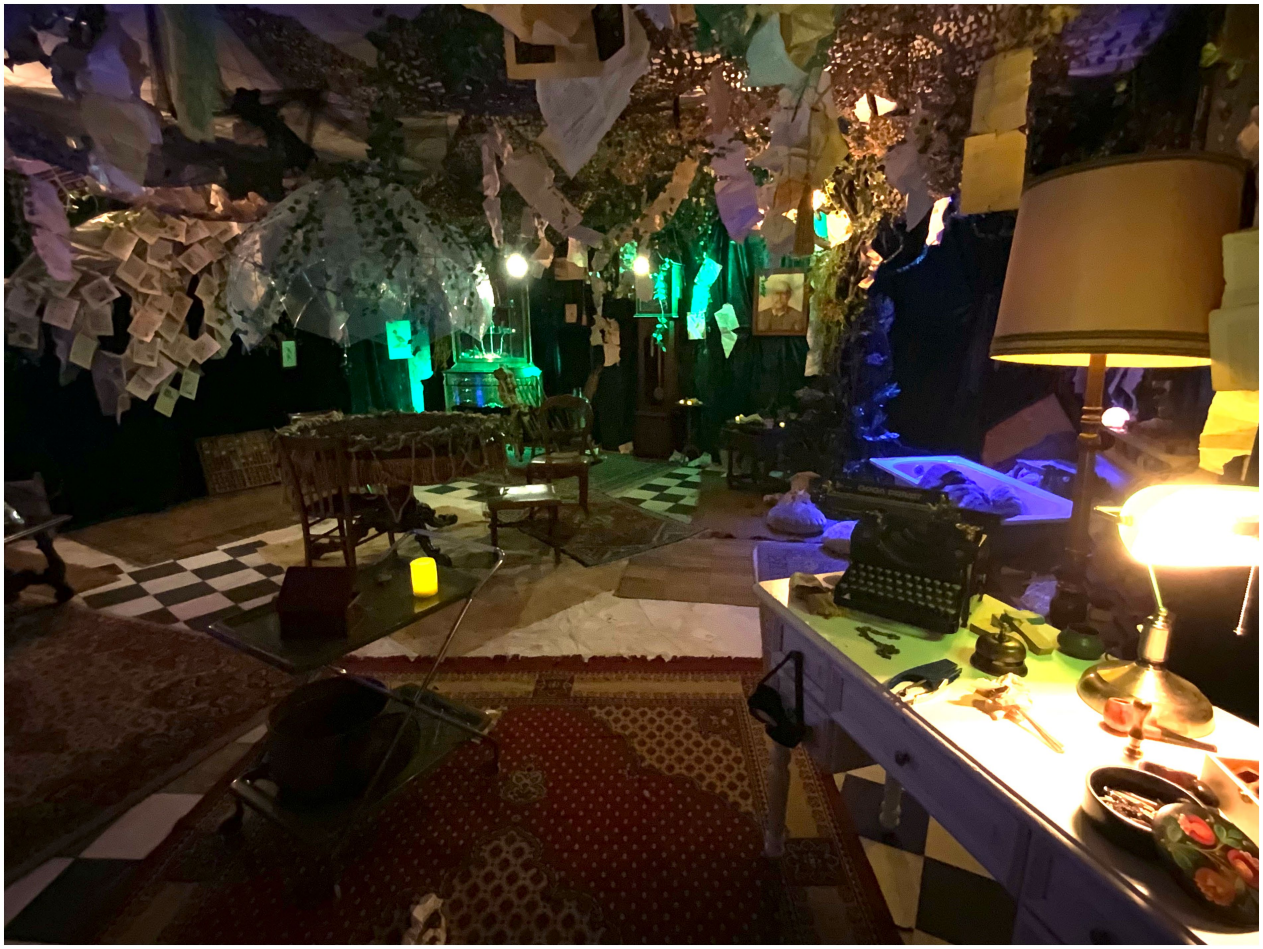
‘The Guest’ set by Donato Marrocco, Martina Giannico, and Gianluca Pozzo, Project xx1, 2022. Primary School ‘Rosetta Rossi’, Rome, Italy. The set-up was created in a traditional classroom.

While these organisations develop immersive learning projects according to different frameworks, objectives, and targeting different kinds of students (e.g., of different ages) in different schools, the strategies at the base of these interventions are somewhat similar. All of these projects are based on the use of a fictional narrative that turns the pupils into the protagonists (or co-protagonists) of a bespoke adventure, created with the collaboration of teachers, headteachers, and school staff, hinged on the materialisation of particular objects or installations in the school itself. In this way, these experiences introduce an element of adventure (or ‘magic’, as Peter Higgin from Punchdrunk Enrichment describes it; Higgin, 2018b), born out and fitting into the everyday reality of schools (Higgin, 2018a), open to student participation, without role-play: the pupils are usually not assigned any specific role other than themselves (Osborne, 2023). The participation is usually facilitated by the realism and detail of the stories and installations: even if the experience is based on fictional characters and stories, even including ‘magical’ and supernatural elements, the implementation features detailed lifelike interactions, painstakingly built sets and props (see Figure 1), and a believable integration with the ordinary school routine.

Creating these kinds of learning environments offers the possibility of an engaging experience, created to fulfil learning needs, and open to different types of active participation. These experiences have been especially precious in deprived areas and to foster the inclusion and participation of children and youngsters with learning disabilities (Ogwuegbu et al., 2023).

The narratives at the base of these educational projects provide an alternative way to deliver the school curriculum (Bowtell, 2015), along with the active practise of soft skills including teamwork, creativity, problem solving, critical thinking, discussion, and emotional intelligence. The apparent ‘rebellious’ streak of these kinds of propositions allows for pupils who tend to challenge traditional schoolwork to participate in, without feeling inconsistent (Bowtell, 2015). There is something substantially different between doing grammar exercises on a school desk or helping a genie (‘Arabian Night Adventure’, Coney, 2015), discovering a clandestine radio broadcast studio inside the school (‘Changing voices’, Project xx1, 2022), or helping two characters escaped from a book to return safely inside its pages (‘A small tale’, Punchdrunk Enrichment, 2016).

In Immersive Learning, agency and narrative are deeply linked: the learners’ possibility to interact with what is happening is scripted in the process itself (see Roth, 2019, for an analysis of an interactive piece aiming at *transformative learning*). Sometimes, in projects based on interactive theatre, the learning space is co-created with the learners themselves (Siciliano, 2022).

These mechanisms generate a learning process which can be included in the framework of Kolb’s Experiential Learning. Kolb (1984) provides a definition of learning that integrates the main ideas of Dewey, Lewin, and Piaget: ‘Learning is a form of transformational process whereby knowledge is converted into experience’ (Kolb, 1984, p. 38). In Kolb’s explanation of learning, two notable aspects emerge: the significance of the learning process itself; and that knowledge undergoes continuous creation and recreation through the process of transformation. Despite being one of the most influential models regarding experiential learning theory, Kolb’s approach has been criticised (e.g., Garner, 2000). Morris (2020), starting from a lack of clarity in Kolb’s definition of a *concrete experience*, proposes additional, useful specifics. Originally, Kolb’s framework includes four Learning modes: ‘This process is portrayed as an idealised learning cycle or spiral where the learner ‘touches all the bases’—experiencing (CE), reflecting (RO), thinking (AC), and acting (AE)—in a recursive process that is sensitive to the learning situation and what is being learned’ (Kolb, 2015, p. 51). Revision of Morris (2020), specifies that experience should be *contextually rich* (whereas Kolb idea of experience was not specifically situated in contexts), reflection should be *critical,* thinking should be *contextual-specific*, and acting should be a *pragmatic test* of what is learned in context. As explained in detail throughout this paper, Immersive Education includes all these specifics, following all phases described by Kolb, along with the specifics proposed by Morris.

## 4 Immersive education key-concepts and their psychological bases

The development of a Project xx1’s Immersive Education project stems from some basic principles that inspire and instruct all of its phases, from planning, through implementation, to evaluation. Because of their pervasive influence throughout all the phases of an Immersive Education intervention, we prefer to think about these points as key-concepts, working synergically in interaction with each other, guiding the whole process. The four key-concepts at the basis of Project xx1’s approach will be described, along with a brief explanation of the psychological mechanisms that can account for their effectiveness.

### 4.1 Motivation trigger

A crucial element in education and school performance has traditionally been motivation (Ryan and Deci, 2020). In pedagogy and psychology, motivation is usually divided in *intrinsic motivation*, the drive to engage in activities ‘for their own sake’, for their inherent interest and enjoyment (Deci and Ryan, 2000), and *extrinsic motivation,* the drive to engage in activities because of external reasons, not inherent in the activities themselves (e.g., for rewards, approval, or instrumental reasons). While educational endeavours are often based on reward-and-punishment systems, school achievement has been demonstrated to be connected mainly to intrinsic motivation (Froiland and Worrell, 2016; Taylor et al., 2014). As Ryan and Deci (2017) point out, intrinsic motivation is likely responsible for the preponderance of human learning across the life span, as opposed to externally mandated learning and instruction.

Immersive Education revolves around the key-concept of being a motivation trigger: Every element of its implementation is developed as an opportunity to enhance both intrinsic and extrinsic motivation. Extrinsic motivation is stimulated by an articulated justification for all the activities that should be performed: Every single proposition to the students is presented as necessary for specific reasons, consistent with the narrative. Specific examples may be: helping someone asking for assistance, solving a problem to unfold a sequence of events, or investigating a mystery. Moreover, every activity is followed by a reinforcement—a specific effect that propels the narration forward, rewarding the efforts of everyone. School activities are often justified by the generic need to acquire competences to play a role in society, to learn skills for future work, or simply invoking some broad moral obligation (e.g., ‘it must be done’). However, many pupils are not stimulated by these kinds of justifications, with the result of making them feel that several school-related activities are pointless. By using narratives to justify activities, Immersive Education offers very different, concrete and straightforward extrinsic motivations that make sense in the context immediately surrounding the participants.

Intrinsic motivation is stimulated by including many different forms of activities in an intervention, spanning from creative tasks to game-like interactions, from group collaborations to individual work. Moreover, every activity is usually achievable in different ways, in order to meet the individual preferences of diverse students. This feature makes Immersive Education projects generally more inclusive than usual school work: they offer a wide range of activities, more prone to capture the interests and the potential of pupils with learning difficulties. The *provision of choice* has been highlighted as an aspect crucial to boost ownership of the activities and greater autonomy: when students experience a *sense of choice* their intrinsic motivation is enhanced (Ryan and Deci, 2020, p. 3–4).

One of the most influential theories of motivation in recent literature is the *Self Determination Theory* (SDT) (Ryan and Deci, 2017). The SDT points out 3 basic psychological needs (Ryan et al., 2019), believed to be essential to support learning processes: autonomy, competence, and relatedness. Immersive Education is designed to contribute to the fulfilment of all three needs. *Autonomy*, as the sense of initiative and ownership in one’s actions (Ryan and Deci, 2020), is supported by making the pupils one of the main characters of the experiences included in an Immersive Education intervention. The choices offered by the multiple ways in which something could be achieved (e.g., creative work), and the fact that all these ways are equally welcome in an Immersive Education project, are functional towards a feeling of interest and value in the experience. *Competence*, defined as the feeling of the possibility that one can succeed and grow, is provided through narrative rewards (e.g., every achievement propels the story forward) and the balance between inclusive and challenging activities, appropriate for the specific age-group. Lastly, *relatedness*, concerning a sense of belonging and connection, is touched by the personal involvement and the fact that Immersive Education experiences are largely group adventures, fostering collaboration based on mutual respect, inclusion, and care.

Immersive projects also specifically stimulate the *Sense of Agency* (Hon and Yeo, 2021), the feeling that something is under our control, based on how the outcomes of an action follow what we expect. The Sense of Agency has been recently connected with better attentional and mnemonic performances (Zou et al., 2023). Immersive projects take advantage of these effects by designing its experiences and activities to give the participants the sense of being the main agents of the events, valuing personal initiatives, ideas, and actions. This is achieved by making the participants play a central role in the story (Osborne, 2023). In other words, the participants are put in a position where they can clearly perceive a sense of *responsibility*—e.g., something is asked from them, and they are the only ones that can provide help—that makes them feel central to the unfolding of the experience.

Whereas Immersive Education often integrates game-like activities, its approach is different from pure *gamification* (Sailer and Homner, 2020). Gamifying an experience means using game design elements (such as points, badges, or leaderboards), in non-game contexts. While gamification is usually hinged on contingent and performance-based rewards, Immersive Education is an approach more akin to *game-based* learning (Qian and Clark, 2016), where games are used as problem-solving spaces and challenges, thus rewarding more basic needs such as autonomy, competence, and relatedness (see also Krath et al., 2021). Crucially, in Immersive Education, learning is the goal, but not the focus (Ryan and Rigby, 2019): every activity and learning process is instrumental to explore the situation at hand, to unravel narrative content or to live through an experience; in this sense, every substantial learning is *incidental* from the pupil’s point of view.^4^ This strategy has been repeatedly proven to be more effective than making the learning process the focus of a gamified activity (Ryan and Rigby, 2019, p.168–170).

### 4.2 *Dramatic structure* and flow

A compelling story is a crucial backbone for any Immersive Education project, as for other kinds of educational projects based on participation (e.g., Slota and Young, 2017). The main storyline is created taking into account several constraints: the specific learning objectives; the resources at hand in terms of actors/educators, technical staff, scenography; the duration of the project; the availability of spaces; the age and background of the participants. Moreover, the specific narrative should be based on interactive elements: the participants should be involved in it from the start, often strategically being the agents that are able to propel the story forward. Triggering a sense of responsibility, as mentioned above (see § 4.1), is achieved with a dramatic mechanism where the participants are made the protagonists or co-protagonists of the events (Osborne, 2023). However, in this mechanism, the participants are not playing a role other than themselves:^5^ Immersive Education creates extraordinary circumstances around them, to stimulate them to experiment their own behaviour and initiative while living through unusual events (*ibidem*).

The extraordinary elements are a crucial contribution to the stories used in Immersive Education interventions and their balance is extremely relevant to enhance the engagement of the pupils. Every Immersive Education activity usually starts with an incursion of extraordinary inciting events in the daily routine. These elements are capable of opening the landscape of possibilities, elicit reactions and initiative from the pupils, and create a new ‘space’ for interaction. Nevertheless, these elements should be appropriate for the age and background of the participants. For instance, when working with younger pupils, the use of supernatural elements (e.g., aliens, parallel dimensions, magic, see Appendix 1) is potentially effective, while older participants are more probable to be engaged by dynamics and forces more connected to real-life experiences (e.g., unlikely, but still possible, events, see Appendix 2). As Slota and Young (2017) pointed out, a narrative should not only be created to convey a message (Narrative-as-Designed), but it should also take into account the response and interpretation of the recipients (Narrative-as-Perceived). The understanding of this bi-directionality is not only essential to establish a creator-recipient relationship, but is crucial when the story develops with the active involvement of the participants themselves. Since the participants must be immersed in a narrative, the narrative should be detailed, life-like and resonate well with their own personal real-life experiences and emotions. In this sense, the dramatic flow of the events should be built using all the traditional narrative strategies used in writing (*cf.* Herman, 2009)—e.g., an Aristotelian 3-acts structure, a triggering event, different characters (either present or evoked), antagonists, a climax, resolution, open ending, plot twists, surprise revelations, etc.—to carry the participants themselves through a cognitive, physical, and emotional journey. The flow of events should be arranged following *necessity* and *logic*: Instead of organising what happens as a function of the learning process alone (e.g., explanation followed by exercises), the elements should be concatenated in such a way that every event is the cause (or contains the seed) of the subsequent ones. The sequence thus built should be *meaningful* and able to be understood as a whole story. The inspiration for the narrative can come from books, movies, comic books: it can be faithful to the original, modified, or created as a completely new narrative.

Narrative messages have been found effective in influencing individuals’ beliefs, attitudes, and behaviours (Ma and Nan, 2018a, 2018b; Ma et al., 2018; Murphy et al., 2011; So and Shen, 2016). This effectiveness arises from individuals’ engagement with the narrative as a whole and with its characters (Green and Brock, 2000). The literature suggests two main mechanisms for narrative influence: narrative involvement and character involvement (Green and Brock, 2000; Moyer-Gusé, 2008; Slater and Rouner, 2002). Narrative involvement refers to individuals’ focus on story events, leading to temporary disconnection from the real world, while character involvement refers to the connection and resonance we may find with specific characters of a story (Busselle and Bilandzic, 2009; Green and Brock, 2000; Slater and Rouner, 2002). Green and Brock (2000) define *transportation* as a unique mental process involving the integration of attention, imagery, and emotions. This concept aligns with narrative presence, including losing self-awareness and mentally entering another world, similar to spatial presence from immersive media literature (Tukachinsky, 2014).

The interactive elements of the narrative should be conceived as spaces open to accommodate different responses, initiatives, and creative ideas, without disrupting the general direction of the story. The ability to do so while maintaining an organic flow of events is the primary objective of the Experiential Theatre’s procedural author (Murray, 2017; White, 2013).

One open question is if the participants of an Immersive Learning experience do actually believe the events of the experience as true (Osborne, 2023). While some accounts suggest some youngsters do come to believe in the production as real (Bowtell, 2015), others claim that the participants ‘perform’ belief together as a group in order to unlock the affective power of the experience (Colvert, 2018, p. 58). In Project xx1’s experience, every unusual event will always stimulate a certain amount of disbelief: the way in which this disbelief should be dealt with depends on the learning objectives of the project, the participants’ age, and several other factors. Project xx1 applies specific strategies when such natural disbelief appears: namely, instead of directly focusing on the actual truth of what is happening, it is possible to interrogate the participants about what they would do *if* the unfolding events were actually true, or to invite them to focus on the common experience they are actually living together as a group, concentrating on the actual reactions and emotions they feel in that precise moment. This also works as a covert invitation to ‘play along’ with the adventure they are facing.^6^

However, calling for a simple ‘suspension of disbelief’ may not be sufficient since, as Murray (2017, p.107-109) points out, when we get engaged in a narrative, we are *actively creating belief*. The dramatic structure of an Immersive Learning activity should call for such availability, should generate the enthusiasm for an active creation of belief, adjusting its content to the specific context and target group.

The dramatic structure and flow of an Immersive Education project should be able to facilitate and foster the participation in the activities, to resonate with the learning objectives, to stimulate curiosity, and to provide a captivating track for the unfolding of the events.

### 4.3 Touching the *self*—involvement, personalisation, and embodiment

It has been demonstrated experimentally countless times that when something involves our *self*, we are better at processing it than when it does not: this is known as the *Self-Reference Effect* (Beggan, 1992; Belk, 1988, 1991). The simple perception of owning something makes it significantly easier for us to remember it (the *Mere Ownership Effect*; Cunningham et al., 2008). Other effects are connected with better memories for self-chosen materials or activities (the *Self-Choice Effect*; Takahashi, 1989; Takahashi and Umemoto, 1987) or better value attributed to things we contribute to create personally (the *IKEA Effect*; Norton et al., 2012). For example, in a recent research study conducted by Leshikar et al. (2015), participants were asked to evaluate either the personal significance of positive and negative adjectives or the semantic meaning of these terms. The study revealed that individuals exhibited better memory recall for items judged in relation to themselves compared to those evaluated solely based on their meaning. Moreover, participants showed a higher retention of episodic details when items were judged in relation to their own selves rather than for their semantic significance. These findings not only suggest an enhancement in overall memory recall but also emphasise the increased integration of perceptual detail in memory through self-referential processing. Research in the field of perception has also demonstrated a similar effect. For instance, Sui et al. (2012) implemented a task where participants had to rapidly judge whether shape/label pairs corresponded to a previously learned relationship. Initially, participants learned to associate simple shapes with labels representing the self, a familiar other, or an unfamiliar other. Following this learning phase, participants completed trials where they were presented with a shape and a label for a brief period and had to judge whether they matched the learned relationship. Sui and colleagues found that participants were faster and more accurate in perceiving matches involving the self-compared to matches involving unfamiliar others. This effect, replicated multiple times (e.g., Sui and Humphreys, 2015), underscores the influence of self-representation on perception.

Other studies indicate that the Self-Reference Effect (SRE) is also influenced by the emotional valence of the stimuli. Specifically, research has shown that positive information, such as trait adjectives like ‘kind’ or descriptions of behaviours indicating care for others, is more effectively remembered when processed in relation to oneself compared to negative information, such as adjectives like ‘dishonest’ or descriptions of behaviours indicating neglect of obligations to friends. This difference in recall is particularly noticeable when information is self-referenced, as opposed to being referenced to another person or processed for its general meaning (Denny and Hunt, 1992; Sanz, 1996; Sedikides and Green, 2000). The impact of emotional valence on memory for self-related information is likely driven by self-enhancement motives, reflecting individuals’ preference to process positive rather than negative information about themselves, such as information indicating competence, likability, or attractiveness (Taylor and Brown, 1988). In other words, when individuals encounter new self-referential information, they tend to prioritise positive aspects and may overlook negative aspects, resulting in better retention of positive information compared to negative one. Thus, personal involvement is undoubtedly capable of enhancing our cognitive and emotional processing.

Personal involvement is a key-process in Immersive Education: what is happening is happening *to* the participants (e.g., as opposed to *in front* of them), it demands *their* attention, *their* skills, *their* actions. Storywise, this is exactly what it means to make the participants one of the main *protagonists* of the narrative—without their involvement, nothing would happen. Their participation is constantly rewarded by the consequences of their actions—these consequences are well visible, tangible, and significant. The fact that results in an Immersive Education project are achievable in different ways, allows for everyone to be able to choose the course of actions that better suits them. This makes the participants’ selves absolutely central in the process, personalising the experience, adapting to the circumstances as they see fit. When the participants feel that what is happening is about themselves, they are much more available to face difficulties, show initiative, and be creative.

Personal involvement is especially intensified in Immersive Education through a constant stimulation of the body. The props used are real objects that can be touched, manipulated, and explored; smell and taste are evoked or directly stimulated; visual images and rich sounds are used; several sections are based on kinesthetics, inviting participants to move, explore, or physically behave in specific ways (e.g., move silently). This multisensory stimulation is accomplished through specific activities, but its primary source are the installations that house the core elements of the interventions. Space is transformed through a detailed setup, enriched by specific lighting and sound, to shape the experience. It is usual for participants to link the most captivating elements of the projects exactly to the set up spaces.

Presenting stimuli across different sensory modalities at the same time shows enhancements in memory (Delogu et al., 2009; Thelen et al., 2014), learning (Shams and Seitz, 2008), attention (Santangelo and Spence, 2007; see also Lunn et al., 2019), reaction times (Hecht et al., 2008), and perception (Pannunzi et al., 2015). Multisensory stimulation showed enhanced experiences for users of immersive technology as well (Melo et al., 2020). The use of different sensory modalities has been found to be especially effective to amplify emotions (Jeong et al., 2011).

Physical and motor experiences have been found to be essential in shaping our mental states and processing, to the point that in psychology it is now common to refer to *embodied* (Shapiro, 2019) or *grounded cognition* (Barsalou, 2008). This approach studies exactly how bodily experiences can influence cognition and how this connection between body and mind is significant for education (Shapiro and Stolz, 2019). The Immersive Education methodology is crucially based on continuous and varied physical experiences and multisensory stimulations, to offer several opportunities to affect the mind through the body.

### 4.4 Multifaceted, *continuous engagement* and accessibility

School activities can often be repetitive and tiresome: the exercise of competences, if not constantly varying in form, can easily turn an initial captivating interest into deep boredom. Several techniques have been developed to try and keep pupils engaged: e.g., connecting teachings to the real world, intercept students’ interests, using mixed media, offering opportunities to develop and share students’ ideas, and the aforementioned gamification (Krath et al., 2021). School engagement is seen, among other things, ‘[…] as an antidote to low achievement, high levels of student boredom and disaffection, and the high dropout rates in urban areas’ (p. 305, Fredricks et al., 2005).

School engagement has been described as a multidimensional construct providing a rich picture of learning, including behavioural, emotional, and cognitive aspects (Fredricks, 2011). *Behavioural engagement* is related to attendance, participation, and positive conduct; *emotional engagement* focuses on the extent of positive and negative reactions to the school, teacher, activities, including feelings of belonging and valuing of school (e.g., Voelkl, 1997); *cognitive engagement* focuses on the level of investment in learning, including being purposeful and willing to exert the effort necessary for the comprehension of cognitively complex ideas and the acquisition of difficult skills.

Immersive Education methodology is designed and articulated to enhance all three kinds of engagement. All these kinds of engagement are stimulated *in primis* by the involvement in the narrative, not only as passive recipients but as co-creators.

This practise hinges on the concept of *narrative co-construction*: inviting, implicitly or explicitly, participants to co-create a narrative by putting forward ideas, providing solutions, or simply by welcoming and sharing their reactions to it. The outcomes of co-construction may be unpredictable but they support creativity, critical thinking, and problem solving in ways not typically seen (individually or collaboratively) in traditional instruction (Slota and Young, 2017).

More specifically, behavioural engagement is fostered in Immersive Education by providing a wide range of activities: pupils are invited to investigate, share ideas, create original materials, collaborate together, solve problems and puzzles, move, etc. All these activities are planned (and justified) according to the developments of the narrative and are organised avoiding repetitions and dead time. Emotional engagement is one of the main aspects of Immersive Education projects: all the activities are orchestrated with the narrative in order to articulate an effective journey for all participants. The experiences are strategically populated with several elements eliciting positive emotions such as surprise, happiness, intrigue, and fun, as well as some parts evoking more challenging emotions such as suspense, misgiving, and apprehension. The involvement of emotions necessitates the ability to welcome and process them by the personnel involved in every project: the actors/educators that give life to Immersive Education must be carefully trained to manage the emotional responses of the students, in order to integrate them in the experience flow. This ability includes giving opportunities to address emotions, to share them, to process them individually or collectively, and to regulate the activities accordingly, up to the point of suspending them to work on specific emotional responses.

Finally, cognitive engagement is obtained by specific dynamics, such as the use of mysteries to be uncovered, creative opportunities, the feeling that the events are dependent on participants’ decisions and actions (even if the result is pre-planned to an extent; see also Osborne, 2023). The cognitive aspects are also particularly stimulated by the interaction between participants and with the characters. The participants are often put in situations where they need to understand a character’s intentions, feelings, or behaviour, even when the characters themselves are trying to hide them. This represents an opportunity to practise interpersonal cognitive skills such as intuition, interpretation, communication, empathy, mentalisation, and perspective-taking (e.g., Carlson et al., 2013; Leslie et al., 2004).

The focus on engagement allows also for an increased accessibility of the activities. The variety of activities, the possibility to value the creative participation of pupils, and the possibility of participating in different ways to the narrative are all features that facilitate the participation of everyone. Moreover, the work with a class often is carried out in small working groups (e.g., creative activities, investigations on clues, exploration of complex environments, etc.), hence fostering the inclusion of pupils with special physical, emotional, or cognitive needs.

## 5 Designing the learning experience and implementing the key-concepts

**Figure 2:**
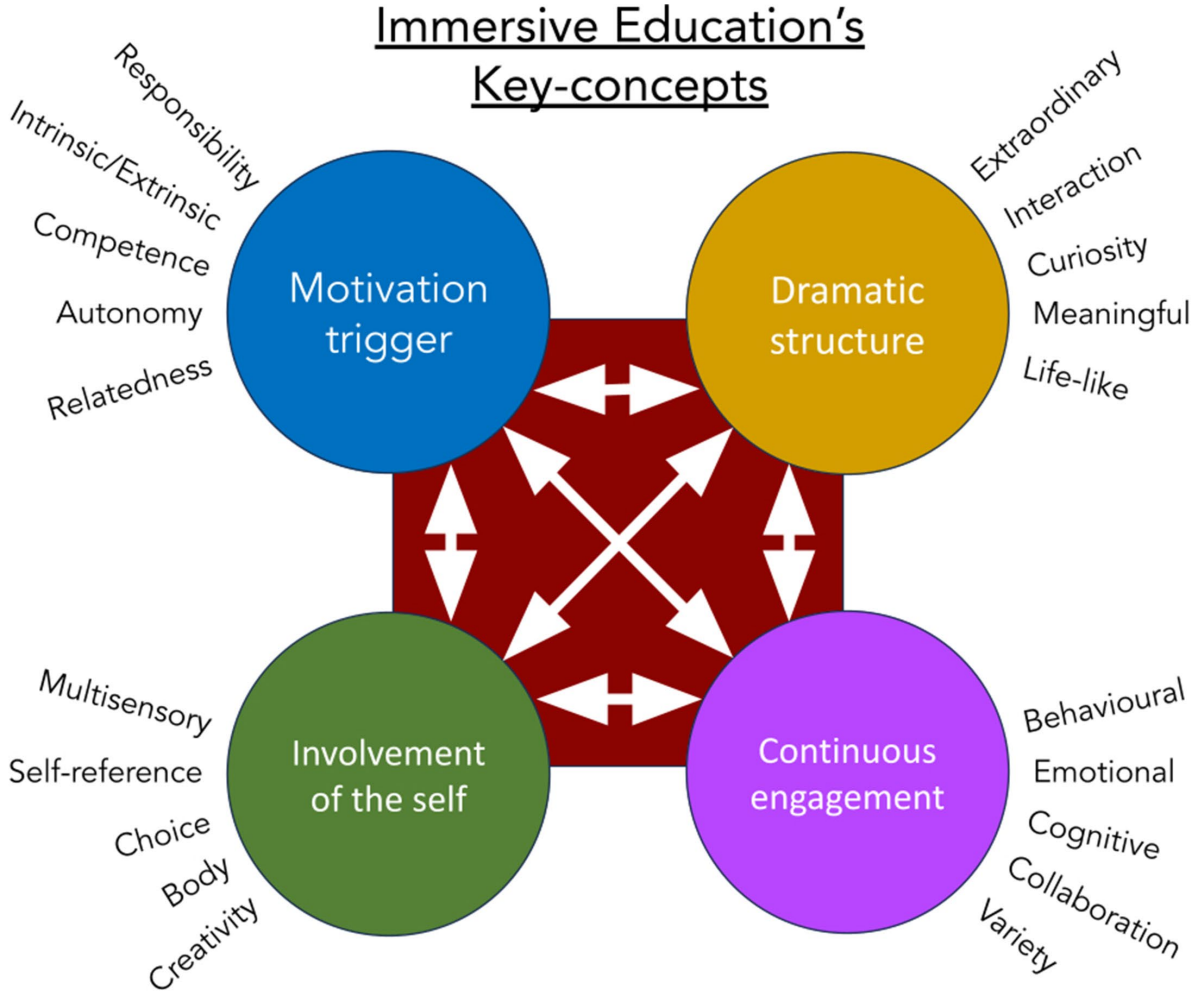
The four key-concepts of Project xx1’s Immersive Education methodology. The arrows indicate the necessity of interconnection and synergy between the concepts. This deep interconnection extends also to the specifics of every concept: Many of these specifics are connected with each other (e.g., Autonomy, Choice, and Meaningful; Collaboration, Relatedness, and Interaction; Curiosity, Creativity, and Emotional, etc.).

All the key-concepts at the root of Immersive Education (see Figure 2) are deeply connected and support one another. However, all of these ideas risk amounting to nothing if they are not implemented skillfully. The following section provides several examples of how the concepts illustrated above can be turned into significant learning experiences.

### 5.1 Setting the learning objectives and the topic

Every planning of an Immersive Education intervention begins by setting specific curricular and extracurricular learning objectives. These objectives are set beforehand by the authors of each project, in close collaboration with the headteacher, the teachers, and other teaching staff, according to the general learning programme and to specific class, or sometimes even individual, needs. Immersive Education projects are usually custom-made or adapted to specific contexts; moreover, the definition of precise requirements is of great benefit for the activities, as it helps the whole operation to fit in the specific school context by focusing on appropriate goals.

The dynamics put in motion by this methodology are apt to tackle with both curricular and transversal competences. Contentwise, the specific themes and topics touched by a bespoke Immersive Education project can be not only akin to school subjects, but they can often exactly overlap with them. The specific subject elements can be embedded in the narrative, along with sensible justifications for their presence. The curricular elements can thus be an essential part of the dramatic structure and flow. On the other hand, the mechanics of interaction and co-creation are particularly appropriate to exercise and practise soft skills. Transversal competences such as negotiation, teamwork, communication, discussion, and problem solving are by definition at the heart of the methodology: The narrative comes alive exactly by fostering interaction and collaboration with the story elements and between the participants. More specifically, thanks to its essential features and dynamics, Immersive Education seems especially suitable to enhance the Personal, Social and Learning to learn Key Competence, as described in the European LifeComp framework (Sala et al., 2020). Finally, more domain-general cognitive and affective abilities such as logic, emotional intelligence/regulation, and creativity are also addressed through this methodology.

To provide an example, *The Guest* (2021, see Appendix 1) was created for 3–4 graders starting from several learning objectives: these included the curricular ‘learn to recognise and compose different types of texts’ and the transversal skills ‘communication’ and ‘teamwork’, among others. Moreover, the classes included several pupils for whom Italian was a second language and their skills in reading, writing, and communicating verbally in Italian were still developing to reach the level of their fellow students. These specific needs were used to define several elements of the narrative and the activities. Specifically, the choice of making one of the main protagonists of the story a *gardener of words* allowed for a metaphorical approach able to deliver some core principles such as *words are nourishment*, *words need care*, *words can be* ‘*grafted*’ *to create neologisms*, *words are powerful*, etc.

Moreover, the activities included several moments when the pupils were invited to work in small groups and then communicate to the others the results of their common work. For instance, among the first activities there was the analysis of several peculiar objects found by the school janitor: small groups were formed, to observe them closely and discuss the meaning of one object for each group. The objects were carefully created to hide common meanings, so that when the groups shared their findings, their discoveries would be confirmed by others. To fulfil the curricular objective, different types of texts were used for activities throughout the project: letters, forms of poetry, word games, etc.

Every time a specific project must be repeated for different pupils, an adaptation is planned by working initially with the teachers and the teaching staff. For instance, in the case of *The Guest*, the activities needed to be attuned to the age and level of the participating classes: The activities were made more challenging for fourth graders and less so for third graders, along with changes in the specific forms of text used, their length, etc. This strategy is implemented to stimulate *motivation* and maximise the *engagement* (see § 4.1 and 4.4).

Every project may be characterised by a general *topic*, an issue or issues around which all of the activities revolve. However, differently from other educational projects, while the topic surely plays a central role in the narrative, it is not immediately addressed directly, but rather it emerges during the activities. Ideally, the topic should be uncovered by the participants themselves, during the activities, as a central pivotal point of what is happening. For example, in *Changing Voices* (Appendix 2) all the project revolved around the topics of *Bullyism* and *Inclusion*. Nevertheless, these topics were not introduced explicitly before the third meeting, when the participants discover the secret broadcasting station. While hints about unfair treatments and exclusion were disseminated during the first two meetings, it was only when they uncovered the secret radio that these issues became gradually evident.

### 5.2 Writing, composition, and interactive elements

On the basis of the established learning objectives, the narrative and the general structure of the activities can be planned. The writing process is faced starting from the idea that the narrative should be appropriate for the age and cultural background of the participants (see § 4.2) and should be interactive in all of its elements. This calls for the skills of *procedural authors*: writers competent in educational matters, able to craft a narrative that allows for active discovery and participation instead of passive fruition. The narrative elements should stimulate curiosity and should allow for a significant role for the participants. This significant role is created by giving the participants a protagonist role in the events, by welcoming certain degrees of freedom for interactions, without allowing for this freedom to derail the main storyline, and finally by planning several moments of co-creation in the narrative.

As an example, in *Changing voices* (2022, see Appendix 2) the participants were allowed to look for information about the founders of the secret broadcasting radio. This information was scattered all around the secret broadcasting studio and included the founders’ identities, their interests, their ideas, their physical appearance, their roles in the broadcast, etc. The search was mostly left to the responsibility of the participants and, if some essential information for the narrative should have not been found through their effort, one of the actors/educators involved would have filled the missing information by passing it as their own discovery.

The elements allowing for a creative input during the experience are another essential element in the process. Every participant should feel that the experience, in specific moments, welcomes their own contribution using their own ideas and their own style: by reflecting real-world dynamics, the effectiveness of their contribution will be tested during the application of their ideas by the situations themselves or by the other participants. The goal of the writing itself and of the actors/educators during the implementation is to maintain a solid chance for everyone’s initiative: all participants must be continuously included and have a shot at creating materials, solutions, or proposing ideas, no matter if their previous contributions were not effective or did not meet the favours of the others. This specific aspect aims at maintaining a continuous engagement in the activities for everyone (see § 4.4).

For instance, the climax of *Changing voices* (2022, see Appendix 2) was represented by the broadcast of a radio programme created and performed by the participants. Every participant was asked to contribute creatively to the programme, following the sections that constituted the original broadcast of the founders of the secret radio. This allowed for proposing comments, anecdotes, opinions, and even music to be included in different sections of the programme. While everyone was encouraged to perform in the broadcast as a speaker, by including both individual and choral moments, this kind of participation was not mandatory, as some participants’ contribution could be also simply as authors of the programme itself.

Lastly, keeping an eye on the whole structure is essential in writing an Immersive Education intervention. The sequence of events should be carefully scripted, ensuring an alternance between different kinds of activities, with carefully planned timing for each one. The procedures should also allow for on-the-fly adjustments by the actors/educators, as the planned activities can be easily deformed by contingencies. The overall result of the writing process should create a compelling unravelling of information and events, featuring mysteries, surprises, plot twists, and discoveries; where participants’ contributions are central and rewarded, culminating in a climax that must be as satisfying as any good story deserves.

### 5.3 Multisensoriality and media

During an ordinary day, we are constantly stimulated by a plethora of stimuli. The life-like character of Immersive Education is achieved with an approach that mimics this richness, by using multisensory elements, spatial installations, and multimedia content. These features implement the principle of personalisation and involvement of the *self* (see § 4.3), by offering the opportunities for an embodied experience. To put in practise this key-concept, all the elements of the experience should have a multisensorial nature. A critical aspect of the ‘immersion’ is the articulation including haptic, visual, auditory, olfactory, and sometimes even gustatory elements, along with kinetic components (e.g., body movement and orientation in space). In order to provide a personal, ‘Immersive’ experience, the more of these channels are used, the better.

Space itself is shaped and populated by many of these elements, creating explorable installations with a relevant narrative function. These installations are not a simple theatrical ‘backdrop’, but host part of the activities and they act as a relevant part of the projects themselves. They are created to transport the participants into a parallel, extraordinary dimension, where the narrative is able to surround them. This is achieved by using set-ups, props, light and sound design, specific smells. The explorable installations are able to add to the projects a dimension and feeling of wonder and reality at the same time: the spaces are clearly extraordinary (sometimes even ‘magical’, e.g., appearing out of nowhere, from the point of view of the pupils) but they are concrete, real, tangible, and offer a venue where the participants are invited to perform activities, by using the elements they find inside.

The first contact with such an immersive space during *The Guest* (2021, see Appendix 1) is when the participants are invited to explore a large, dark, and apparently empty room with the help of flashlights. Afterwards, this same room will be completely transformed, by turning into the gardener’s home and laboratory (see Figure 1), featuring scenic lights, a soundscape provided by hidden speakers, and specific smells (e.g., provided by a large amount of soil, ready to plant words, in a domestic small indoor greenhouse). The threshold to these spaces is usually marked by a specific way to enter them: the gardener’s home in *The Guest* was accessible through a glowing ‘portal’; the secret broadcast studio in *Changing voices* was accessible through a secret passage through a bookshelf, etc.

The narrative itself is developed over different media: spoken word, written texts, digital content such as audio excerpts or videos, physical objects, drawings, photographs, live interactions with characters, etc. This *transmedial* delivery (see § 1 and footnote 1) has several advantages. Firstly, it fosters *personalisation* (see § 4.3) and accessibility by offering different stimuli, able to meet different attitudes and natural inclinations: some participants will be facilitated by the use of written texts, while others will be more sensitive to digital contents; some will be more attentive to physical objects, while others will captured by speaking and interacting with an actor, and so on. Secondly, since life itself is profoundly transmedial and multisensory, this approach helps in rendering the experience life-like. We are so used to jumping from one stimulus to another that we seldom realise how many sources of information we constantly use in real life: namely, even going to a restaurant involves spatial information (reaching the restaurant), written text and pictures (the menu), interaction with others (the waiting staff, our tablemates), smell and taste (eating), specific codes (numbers to indicate prices or to pay the bill), etc. By providing rich multimedia contents, our *self* gets involved in different ways and multiple levels, encouraging a holistic approach to learning. Lastly, the variation between different sources of information provides an opportunity to renew the participants’ focus and *engagement* (see § 4.4).

### 5.4 Emotional journey

As mentioned above (§ 4.4), emotional engagement is an essential aspect of every Immersive Education project. Shared emotions are able to create bonds between participants, or to create individual long-lasting memories. Emotional experiences are well supported by a dramatic structure and, on the other hand, are capable of making the narrative itself more compelling. Emotions play a crucial role in learning, as numerous studies have demonstrated that human cognitive processes are influenced by emotions, including attention (Vuilleumier, 2005), learning and memory (Phelps, 2004; Um et al., 2012), reasoning (Jung and Hamburger, 2014), and problem-solving (Isen et al., 1987). Moreover, the attentional and motivational components of emotions have been linked to enhanced learning and memory (Pekrun, 1992; Seli et al., 2016). Therefore, emotional experiences/stimuli tend to be remembered vividly and accurately, exhibiting considerable resilience over time. Some studies suggest that positive emotions facilitate learning and contribute to academic achievement, mediated by levels of self-motivation and satisfaction with learning materials (Um et al., 2012). In addition, emotionally enhanced memory functions have been reported in relation to selective attention elicited by emotionally salient stimuli (Vuilleumier, 2005; Schupp et al., 2007).

Since emotions are powerful elements in a life-like experience, their potential must be treated with great care. In Project xx1’s practise, nothing should happen too suddenly: every event that could generate intense emotions is introduced gradually and gently, leaving all the participants time to prepare or adapt to it. Moreover, the intense emotional events are always experienced as a group and are always followed by an opportunity to share and understand what happened and how everyone experienced it. This process allows the participants to connect with their fellows while experiencing emotions, along with the possibility to observe the extent of how emotions are processed by different people. Every perplexity, quandary, or uneasiness is supported through the possibility to express it, explain it, and understand it together. This process is made possible by the close collaboration with the teachers and the teaching staff, since the echo of emotional experiences can sometimes emerge even days after the meetings.

For instance, the first encounter with the gardener in the project *The Guest* (Appendix 1) was created to foster curiosity and a sense of discovery. The participants had to help a man in distress, trying to understand the best ways to support him. Later in the project, finding how their word-seeds grew into word-plants or the demonstration of how the Machine can turn texts into energy, triggered a sense of wonder and excitement. At the end, finding out that the gardener and his laboratory successfully travelled back to their home dimension, by witnessing the now empty room, generated both melancholy and happiness at the same time.

In *Changing voices* (Appendix 2), the final radio (fictional) broadcast was reached after a build-up of several meetings and generated the typical trepidation of a live performance. While the programme was actually only recorded, in the narrative the secret radio was again fully functional and a big ‘On air’ sign lit up as a signal that the broadcast was active. The group was silent, focused, and everyone waited their turn following the script they created: A big release of tension celebrated the end of the performance, with a shared sense of accomplishment.

In the feedback collected after the projects, the emotional journey is usually highlighted by participants and teachers as one of the most intense and memorable features of Immersive Education projects.

### 5.5 Reflection and creativity

Every step in an Immersive Education project must be followed by reflection and abstract conceptualisation, following the inspiration provided by Kolb’s ELT (Kolb, 1984, 2015). It is of utmost importance to provide an appropriate time and space to allow the participants to share and reflect on their personal insights and doubts. These sections provide ‘breathing’ moments that should be embedded in the structure, with a proper integration (e.g., justification) in the dramatic flow. They should not be perceived as suspensions from the sequence of events, but as moments necessary to re-organise thoughts and decide on the following course of action.

One of the essential aspects of the reflection is to allow everyone to participate, with their own thoughts and feelings. Very often, these moments were precious to include participants that were less active during the more intense phases of the activities, or to capture the attention of the less involved, or to clarify something that was not understood by everyone. The moderator of these moments, usually one of the characters leading the experience, must not impose a specific point of view or a fixed interpretation of the events, but should guide the participants through a fruitful confrontation. These are the spaces where the individuals can really express their thoughts and feelings, without any obligation. A special attention during these sections is usually devoted to analyse group dynamics—how the participants feel working with the others, in relation to the events.

Another specific activity often integrated in several kinds of Immersive Learning approaches (see Osborne, 2023) is a creative effort by the participants. The events demand a creative approach to solve a problem or to navigate a specific section of the experience. As an example, in *The Guest* (Appendix 1) the pupils were asked to compose texts for the Machine, or to write a letter to an unknown recipient through an interdimensional mailbox. In *Changing voices* (Appendix 2) they were asked to write a radio programme, following the structure defined by the old founders of the secret radio.

These creative moments can be individual (as the composition of texts for the Machine in *The Guest*) or group activities (as the development of each section of the radio programme in *Changing voices*); while they can be more structured, with some constraints, or unbound, they should be always linked to and justified by the narrative. Every creative endeavour by the participants must be followed by a reward of some kind: e.g., the Machine whirling into life after being fed a textual composition, or the call of an enthusiastic listener after a radio broadcast.

### 5.6 Reality and fiction

An Immersive Education project is an elaborate adventure, created to stimulate participation: it is a work of fiction, used to achieve accessible learning. Despite its fictional nature, one of the most effective features of this methodology is its believability, its complexity, and its life-like features. The theatrical setup—the plot, the script, the characters, the installations, the props, the planned interactive activities—must be developed to integrate perfectly, at least at the beginning of the project, with the daily school routine. This plausibility must be at its maximum at the project inception, in order to facilitate the gradual journey towards more extraordinary features during its development. The activities are thus not presented as school-related work, but as actual events that are happening to the participants. The characters (see § 5.7) with whom the participants interact are usually characters that could be met in a school context, and their presence is not declared as ‘special’ or ‘fictional’. For example, the first character they meet in *The Guest* is a (fictional) school janitor, while in *Changing voices* they are a (fictional) technician and a (fictional) school teacher.

The main reason for this ‘benign deception’ is participants’ motivation and ensuing engagement. If something is presented as an actual event, and not a fictional one, the participants are naturally motivated to understand it fully and prone to engage in investigating what is happening; they value their own initiative and participation as something that can steer the course of events; they apply to their choices and engagement the same rules and determination they apply to real-world decisions; they connect emotionally with what is happening. If we would declare the fictional nature of what happens right away (e.g., ‘let us play together’), this would have the participants treat the activities as any other school-related matter, reacting with known dynamics and attitudes. This initial plausibility soon turns towards magical, extraordinary, or unusual events, giving away the fictional nature of the narrative. However, many participants declare that they were not completely sure about the reality of what was happening, sometimes till the end of the project.^7^

This life-like quality is obtained by a great level of detail in every aspect of the narrative. As an example, in *Changing voices*, the secret radio was founded by four characters around 20 years before, who the participants would never meet in person. These characters were the original founders of the radio broadcast and their example was meant to inspire and guide the participants in their present endeavours. Even if absent, countless clues about the personality of these characters, their appearance, their passions, their problems, and their achievements, were scattered in recordings, props, writings, nicknames, and even caricatures. The 4 characters were developed as all-round people, with their peculiarities, complexities, and emotions. This was done because most of the information about these central characters was to be discovered autonomously by the participants: to ensure a proper reward to a participatory effort, it is important to make sure that every search would find something.

The fictional nature of the project is established with the collaboration of the principal, the teachers, and the school staff. Precise instructions must be followed by all the parties involved, such as the non-disclosure of the narrative as fictional, the restriction of not using the activities as bases for homework or traditional schoolwork (e.g., reports, essays, questionnaires, etc.), and the collaboration on specific turning points in the narrative. When the project is over, the fictional nature of the narrative is confirmed to everyone and the experience of the activities can be used, if needed, as the basis of other schoolwork.

### 5.7 Immersive live educators

All the implementation aspects above must be delivered by professionals who are aware of the principles, the purpose, the characteristics, and the development of an Immersive Education project. There are different categories of Live Educators in Project xx1 interventions: the *inhabitants* (in character), the *bridges* (in character), and the *supporting staff* (as themselves). Each category follows the narrative with specific goals connected to the storyline, and to the educational objectives. Their goals are revealed gradually and are subject to adaptation, following the evolution of the project and the participants’ reactions. The *inhabitant,* being completely part of the narrative, guides the participants into the exploration of it. The *bridge* is usually a character that could belong both to the narrative and the real world, with the task of ferrying the participants from their daily school reality into the narrative. For example, the *inhabitant* could call for help, and the *bridge* could guide the group into the investigation that will lead them to meet the *inhabitan*t in person. Finally, the *supporting staff* are all the other people (school staff, other teachers, older students, etc.) that will put interest and belief in the narrative, supporting its development without being directly involved in it.

All of these roles could be covered by educators, teachers, actors, facilitators, trainers, with more or less acting skills according to the specific role and the competences to facilitate individual and group learning processes. Usually, the *bridge* requires a facilitator, an educator with acting skills, while the *inhabitan*t could have a more pronounced theatrical background along with pedagogical sensitivity. Both the *bridge* and the *inhabitant* require to fully support the narrative through acting and reacting according to the script, while adapting it to the student’s reactions and needs. The *bridge* becomes a point of reference by leading participants from one phase to the other and supporting their journey. To fully play her role, the *bridge* acts as if she does not know anything about the narrative, the discoveries, the tasks and the whole development of the journey.

The required acting skills, essential for both the *inhabitant* and the *bridge*, can be found either in professionals or amateurs with acting experience in interactive theatre. These skills include sensitivity to modulate their physical and vocal expression to engage their audience by creating suspense or mystery, by eliciting excitement or curiosity or by highlighting pathos or fun; to be ready to improvise consistently with their character if unpredictable questions, or other potential issues, arise; to be as authentic and believable as possible in order to facilitate the immersion in the narrative.

Regarding competences in facilitating individual and group processes, Kolbs’ Facilitator Profile suggests that ‘[…] educators help learners get in touch with their personal experience and reflect on it. They adopt a warm affirming style to draw out learners’ interests, intrinsic motivation, and self-knowledge. They often do this by facilitating conversation in small groups. They create personal relationships with learners’ (Kolb et al., 2014, p. 220). In Immersive Education, the *bridge* as facilitator entails to provoke group discussions and reflections with the aim of fostering students’ critical thinking, guiding their insights, organising their actions, or stimulating their creativity. The *bridge* should be able to create profound relationships with learners where emotional skills like empathy, non-judgmental attitude, and an inclusive approach are promoted and shared. They must also support the process without interfering excessively with the participants’ experience and autonomy (see § 4.3). All Immersive Live Educators should also leverage their communication and collaboration skills, enabling them to work with each other and with the school staff external to the project.

### 5.8 The process of creating immersive education interventions

**Figure 3:**
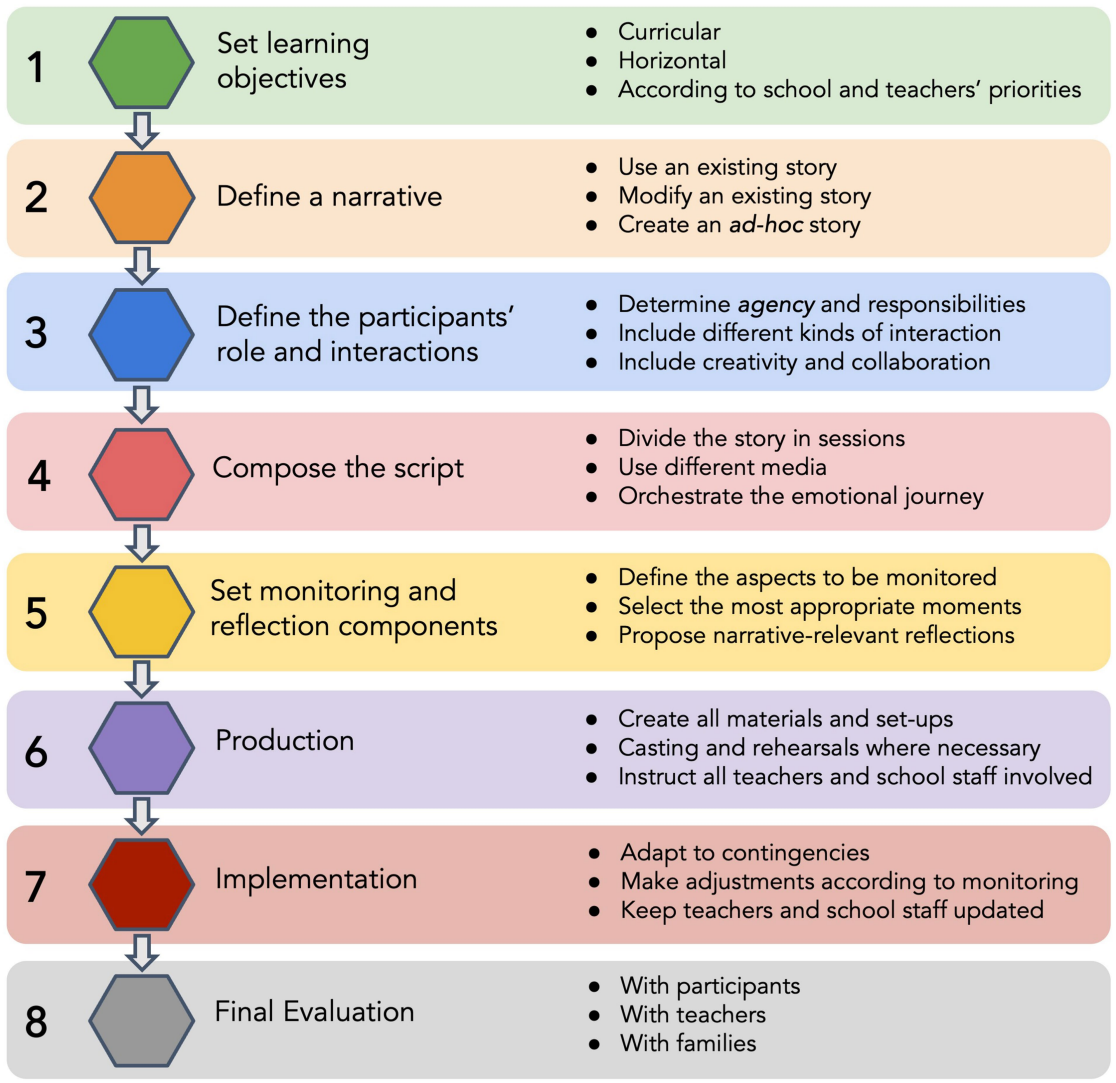
The process of designing, implementing, and evaluating an Immersive Education intervention. These steps may be carried out by the teachers themselves or by external educators, in close collaboration with teachers and school staff.

By following all the implementing strategies described so far, it is possible to organise them in a step-by-step sequence, illustrated in Figure 3. While the steps are rather straightforward, there are some general criteria that need to be kept in mind while moving through the process of creating an Immersive Education intervention.

Firstly, all of these steps should be guided by the four key-concepts: e.g., in defining a narrative (step 2), the *Dramatic Structure* principle may seem to be the main one instructing the choices, but all the other principles should be guiding this step as well. The *Motivation Trigger* principle should be taken into account, by choosing, creating, or modifying a narrative in ways that get to touch significant issues for the specific target group we are planning to work with. Moreover, the *Involvement of the self* principle must be respected, by choosing or creating a narrative where the pupils can play a relevant role. Finally, the *Continuous Engagement* principle must be kept in mind by exploring a narrative that can include a variety of activities, generating a cognitive, behavioural, and emotional engagement.

Secondly, all the previous steps must be considered when working on a subsequent step. For instance, to set monitoring and reflection components (step 5), it is necessary to keep well present the learning objectives (e.g., to make the monitoring and reflection relevant), the specific narrative and the script (e.g., to avoid interrupting relevant story beats), and the participants’ role (e.g., to make the reflections consistent with their interaction with the story).

Thirdly, the most relevant aspects immersive-wise are what is defined in steps 3 and 4. These are the steps where the principles of Immersive Education can really take shape: in defining the interactions and composing the script, we structure how the participants will be involved and engaged in the events. These are also the steps where most of the opportunities for inclusion are created.

Finally, this whole process should rely on the teachers and their knowledge of the target group itself. The teachers are the main source of information about the curriculum, the specific learning objectives, the target group, and the general context of the school/institute. The whole creation can greatly benefit from a specific knowledge of the target group (e.g., the class or classes that will participate in the intervention): their dynamics, the needs of its members, their internal balances and comfort zones, etc. This knowledge should be used to tailor the intervention (or adapt an already existing one) to the specific group, to maximise their learning opportunities.

## 6 Limitations

While the Immersive Education methodology can surely provide unique features to motivate, engage, and include several kinds of pupils, it certainly presents limitations that need to be addressed.

The first general limitation is that Immersive Education projects are usually costly and need a close collaboration between teachers, writers, scenographers, prop-makers, technicians, IT experts, school staff, theatre artists, and educators. This calls for a network effort, involving several kinds of professionals and community resources. This creates obvious limitations in terms of scalability—applying it to several schools at once and with a large number of participants. However, this kind of limit can be mitigated in at least two ways. The first is by considering that the larger costs and intense collaborations are mainly linked to the creation and the first implementation of a new project, while the subsequent implementations need significantly lower resources. Hence, this problem could be partially solved by applying an intervention to as many pupils as possible, to minimise the cost of the most resource-demanding phases, namely the conception and writing (steps 1 to 5, Figure 3) and the set-up (step 6, Figure 3). The second strategy could be to use the principles at the heart of this methodology to develop simpler, teacher-led initiatives (see Osborne, 2023).

The second limitation is linked to the difference that this approach has to traditional school activities. School systems and whoever works in them have been developed around specific methodologies and approaches—everything that is different, needs a considerable amount of accommodation. More specifically, Immersive Education hinges on maintaining a consistent fictional narrative also between the sessions, and it is based on setting up spaces with installations for several weeks (making thus these spaces not accessible for other activities).^8^ Since school institutes are usually accustomed to work in different ways, sometimes the difference between new approaches and the usual daily practise can be problematic.

Finally, another limitation is about the current state of validation of the methodology. While Immersive Education projects have been implemented in Italy and Spain several times, in different institutes, the data gathered in these occasions is currently under analysis. The data has been used locally, within a single project, to monitor and evaluate the process during the implementations to adjust the interventions where and when needed. However, in order to properly evaluate the impact, the effectiveness, and the efficiency of these projects, a more systematic general analysis is necessary. This analysis, guided by the theoretical framework proposed here, would be crucial to modify the methodology according to the actual results obtained across different projects.

## 7 Conclusion and future research

Immersive Education is a methodology characterised by the use of a participatory narrative to facilitate inclusive learning. It can be used in the form of shorter (e.g., 3–4 sessions) or longer (e.g., spanning several months, with more than 10 sessions) projects, integrated with curricular school activities. Different learning objectives can be achieved with it, both curricular and transversal. The approach is based on four interconnected defining principles (key-concepts) and is guided in its implementations by several aspects. This approach, akin to other Immersive Learning techniques (Bowtell, 2015; Higgin, 2018a; Osborne, 2023), is based on Experiential Theatre practises (Lewis and Bartley, 2023).

This methodology is inspired by and fitting in Kolb’s Experiential Learning theory (Kolb, 1984, 2015; Morris, 2020), as it recursively offers opportunities to practise and develop abilities related to the *contextually rich* experience they have lived (CE), the *critical* reflection on it (RO), thinking together as group on the *contextual-specific* phenomenon encountered (AC) and *pragmatically* experimenting in their daily life with this new learning (AE). It fosters a hands-on approach to problems, providing engaging learning opportunities to pupils with different preferences, talents, and peculiarities. While this methodology has been presented here regarding its applications to children and youngsters, Project xx1 has already successfully applied these principles, *mutatis mutandis*, to adult training as well.

As mentioned in §6, one of the first directions for further research is to assess how Immersive Education impacts on learning, inclusion, and engagement, also beyond the timeframe of a specific project, with validated tools and measures. Currently, the data gathered during the period 2021–2024 are being analysed, but even more direct comparisons with traditional teaching methods would be necessary to highlight the impact of the methodology. Future research could thus benefit from between-subject study designs or longitudinal studies that track students’ progress over time and compare their performance with control groups in traditional educational settings. Moreover, these assessments and data collection should happen in ways that do not disrupt the flow of the narrative and do not put the participants in the position of evaluating the experience before it is completed (e.g., with a between-sessions questionnaire, or similar).

Another future direction for research could deal systematically with the different strategies and effects that Immersive Education has when working with learners of different ages. This kind of research should help identify necessary adaptations to ensure Immersive Education is engaging for both younger and older students. Similarly, research should explore how this method can minimise teachers’ workload, by integrating these kinds of projects with existing curricula.

Finally, future research could explore how this framework can be adapted and expanded. For instance, the element of competition could be integrated in the model to enhance motivation and sustaining interest (Cagiltay et al., 2015; Xu et al., 2022; but see also Hanus and Fox, 2015 for opposite effects): competition should not here be intended solely as between different participants, as it can easily be integrated in the narrative as a fictional element (e.g., the group of participants is competing with another fictional group that they will never directly meet). The role of self-expression (see §5.5) can certainly be expanded in future projects, as it can lead to deeper emotional engagement and personal relevance in learning experiences. Furthermore, the dynamics of Immersive Education offer a unique opportunity to explore perspective-taking: understanding different viewpoints can enhance empathy and facilitate deeper learning by encouraging students to engage with narratives from diverse perspectives.

While all these steps will be useful to improve this methodology, Immersive Education already introduces innovative dynamics to organise shared experiences and reflections along a captivating interactive narrative, putting the learner at the centre of the learning process.

## Data Availability

The original contributions presented in the study are included in the article/Supplementary material, further inquiries can be directed to the corresponding author.

## Footnotes

^1^*Transmedia* (Rajewsky, 2002; Wolf, 2015), refers to ‘phenomena that appear in more than one medium without being (viewed as) specific to, or having an origin in, any of them’ (Wolf, 2015, p. 461). In storytelling, it refers to a story spanning across different media: notorious examples are the *Star Wars* and the *Matrix* sagas. In this context it refers to a narrative built across different media such as audio, video, scenography, text, theatre, music, and different kinds of structured activities. Every element adds to the story itself rather than re-telling the same story in different ways.

^2^This term also avoids the ambiguity of the term “immersive” as used by approaches based exclusively on digital technology, such as Virtual or Augmented Reality.

^3^‘In a CPW [Conceptual PlayWorld], the imaginary play usually follows a known fairy tale or children’s book with dramatic moments, that enables the children and teachers to build empathy with the characters (Fleer, 2019). Following the storyline, teachers and children enter an imaginary space full of dramatic problems that need to be solved using conceptual knowledge. Having empathy with the characters motivates children to help the characters’ (Wang, 2024, p.524). While the premise of Conceptual PlayWorld is similar to other Immersive Learning approaches based on narratives, including Immersive Education, this specific methodology has been developed for younger participants, usually pre-school or 1st-2nd graders. This makes many of the aspects connected to the use of fictional aspects, the overall structure and implementation of an intervention obviously very different than working with older participants.

^4^‘People can easily sniff out when someone is trying to manipulate them by mixing unappealing goals and tasks into a game’ (Ryan and Rigby, 2019, p.168). We believe that this kind of observation applies especially in a school context.

^5^While many of the dynamics created in Immersive Education can remind of Role Playing Games (RPG) or, better, Live Action Role Playing (LARP), there are two crucial differences between this approach and role playing. The first, as mentioned in the text, is the fact that the situations do not demand the participants to play a role other than themselves—they are certainly stimulated to participate and react in specific situations, but without pretending to be someone else. The second is that while RPGs and LARPs are games and generally have recreational objectives, Immersive Education’s ultimate goal is to stimulate a learning process.

^6^As one of the children involved in Coney’s ‘Adventures in Learning’ programme commented: ‘I do not think the talking cat is real, but Miss seems to totally believe it, so let us go along with it as it’s better than doing work’ (Bowtell, 2015).

^7^See above, § 4.2, for information about how to deal with disbelief.

^8^It can be very tricky to ask to some teachers to not to use the experience as a basis for traditional school work while the specific project is still ongoing (e.g., asking the pupils to write a report or an essay on a specific experience they had between the sessions of an ongoing Immersive Education project), as this may hijack the believability of the fiction (e.g., if something ‘secret’ happens, it is not believable that you are asked to write a report about it); or ask to school staff that a specific space (e.g., a ‘hidden’ hideout) should not be left open for other classes to use.
