# Supplementary material for: Turning stories into learning journeys: the principles and methods of Immersive Education
Source: Front Psychol. 2024 Dec 6;15:1471459. doi: 10.3389/fpsyg.2024.1471459 (PMC11659684; doi:10.3389/fpsyg.2024.1471459)
Supplement: Supplementary file 1 [file Data_Sheet_1.docx]

Appendix 1

A plot synopsis for “The Guest”, an Immersive Education project implemented by Project xx1 in the primary schools “Largo Dino Buzzati” and “Rosetta Rossi” in Rome.

*The Guest* (2021)

An Immersive Education project for Grades 3-4

Duration: one weekly session (90 mins) for 6 weeks

Participants: approx. 250 students to date

The experience of *The guest* starts when a school janitor (an actor/educator) finds several weird objects in the school and asks if the class is responsible for dropping them around. When the pupils declare their innocence, the janitor shows them what was found and invites the class to investigate to find out what was happening. The investigation reveals, among other clues, the appearance of a weird mailbox in the school. The class is then guided to write a letter asking for clarifications and to post it in the mailbox.

The answer reveals that someone from a parallel dimension is trying to send something in the school, and that the objects were the results of the first attempts at cross-dimensional transportation. This news is followed by the appearance of a weird large jute fabric bag in an empty and dark classroom. The exploration of this classroom with the help of flashlights makes the pupils discover a man in the bag (an actor/educator) - this man is clearly in distress and asks for their help: he is hungry and needs them to provide something to eat. They discover that the man does not eat food, but words, served on coloured and decorated book pages. They leave the man to gain back his strength, and the next time they find out that the empty classroom is now filled with the man’s home and laboratory.

Welcoming them back, the man explains that he comes from another dimension and that he has been sent into exile there by the local dictator, who despises him because of his ideas and profession. After him, the dictator managed to transfer all of his home and laboratory inside their school. The man is a *gardener of words*, and introduces the class to how, in his world, words can be grown, harvested, and used as nourishment. He guides them to plant words, to see how they can grow with his techniques.

When they come back to the gardener’s laboratory they find that their words have indeed grown into word-plants. The gardener is joyous to see that his techniques work well in this dimension and explains the real reason why he was exiled. The dictator wishes to maintain control over his people by reducing the number of available words and the gardener represents a threat, due to his ability of creating/growing new words. The gardener shows to the class his greatest invention, that could be also the key to send him back: The Machine. This apparatus is able to transform written poetry directly into electricity and could work as an energy source for interdimensional travel. After a demonstration of how it works, the gardener asks the class to help him by providing enough fuel in the form of specific forms of poetry, that he will turn into electricity with his Machine.

When they come back, the whole laboratory and the gardener himself have disappeared and the classroom is completely empty. They receive an interdimensional video-message from him, where he confirms that the machine worked and that he is back in his dimension: now he is able to work to change the government’s ideas about words, thanks to their help.

Appendix 2

A plot synopsis for “Changing voices”, an Immersive Education project implemented by Project xx1 in the middle school “Largo Dino Buzzati”, in Rome.

*Changing voices* (2022)

An Immersive Education project for Grade 6

Duration: one weekly session (90 mins) for 6 weeks

Participants: approx. 120 students to date

The project *Changing voices* begins with a memorandum received by the class, where the principal notifies the students that a new initiative will start, based on the installation of an audio system in the classrooms. The system, an odd-looking quiet speaker in a corner, starts to emit static noises and voices, up to the point that the teacher is forced to interrupt the class and call for assistance. A technician shows up (an actor) and guides the pupils in a procedure to “capture” the interference that is causing trouble into small digital recorders. After this, the technician leaves quickly, leaving the recorders with them.

The class is immediately joined by another teacher (an actor), responsible for the installation of the audio system. This teacher appears quite shocked that a “technician” showed up, since no other personnel is aware of the initiative. She invites the pupils to investigate what kind of interference was “captured” in the recorders and together they find out they sound like a radio program and a private message. The teacher leaves to further investigate what is going on.

After a few days, the teacher informs them that she discovered something and invites the class to investigate in the school to look for clues. They find a 20-year old box with old documents set aside by an old school custodian, now retired. They find a map of the school that points to a secret place, but when they follow it, they find themselves stuck in a small library. The teacher tells them that she will try to talk with the retired custodian, to find out what the box and the map are about.

Unfortunately, the old custodian seems to be suffering from dementia, and the teacher shows the pupils a video where the old lady can be seen doing what looks like a dance. The dance is actually a code to find out a secret passage in the library that leads the class in a dark room full of things. There they meet the phoney technician they met at the beginning, who reveals to them that the room they are in is a broadcasting station for a secret radio founded by 4 students more than 20 years ago. He explains that he wanted them to find it, to help his nephew, who is a student in the school and that recorded a message asking for help. They all listen to the full message, recognising parts of it as the fragments they listened to from the digital recorders. He talks about how he is the victim of discrimination and bullying.

The secret radio was created by these 4 students to denounce different acts of bullying they and other friends of theirs were experiencing. They started broadcasting with the help of the old custodian, to raise awareness about bullying and to encourage everyone to speak up, especially if they were passive witnesses of acts of bullying. The teacher asks what they can do to help and the phoney technician says that his nephew’s wish is that they restore the radio and start broadcasting again: he will help them with the technical aspects.

To re-activate the radio they have to explore the room and solve some puzzles and, doing so, they discover more about the 4 founders’ ideas and characters. They find the outline of the programme they used to broadcast from there and the teacher suggests they use it as a structure to create their own programme. In the following sessions, they write and rehearse their programme and they broadcast it with the help of the phoney technician.

After they finished broadcasting, they receive an audio message from one of the original founders, now an adult, that thanks them for their work and tells them that she is moved by the fact that someone found their station and restored it. She hopes they will continue with their fight against discrimination and bullyism.
